# Effects of Drop Jump Training on Physical Fitness in Highly Trained Young Male Volleyball Players: Comparing Maximal Rebound Height and Standard Drop Height Training

**DOI:** 10.3390/sports12120336

**Published:** 2024-12-05

**Authors:** Raouf Hammami, Karim Ben Ayed, Yassine Negra, Rodrigo Ramirez-Campillo, Michael Duncan, Haithem Rebai, Urs Granacher

**Affiliations:** 1 Higher Institute of Sport and Physical Education of Ksar-Said, Manouba University, Tunis 2010, Tunisia; raouf.cnmss@gmail.com (R.H.); yassinenegra@hotmail.fr (Y.N.); 2 Tunisian Research Laboratory ‘Sports Performance Optimization’, National Center of Medicine and Science in Sports (CNMSS), Tunis 1004, Tunisia; haithem.rebai@yahoo.fr; 3 Sport Sciences, Health and Movement Laboratory, High Institute of Sport and Physical Education of Kef, University of Jendouba, Jendouba 8189, Tunisia; ben.ayedk1@yahoo.fr; 4 Research Laboratory (LR23JS01) «Sport Performance, Health & Society», Higher Institute of Sport and Physical Education of Ksar-Said, Manouba University, Tunis 2010, Tunisia; 5 Exercise and Rehabilitation Sciences Institute, School of Physical Therapy, Faculty of Rehabilitation Sciences, Universidad Andres Bello, Santiago 7591538, Chile; rodrigo.ramirez@unab.cl; 6 Department of Physical Activity Sciences, Universidad de Los Lagos, Osorno 5290000, Chile; 7 Centre for Sport, Exercise and Life Sciences, Coventry University, Coventry CV1 5FB, UK; aa8396@coventry.ac.uk; 8 Department of Sport and Sport Science, Exercise and Human Movement Science, University of Freiburg, 79102 Freiburg, Germany

**Keywords:** stretch-shortening cycle exercise, plyometric training, adolescents, neuromuscular adaptation

## Abstract

Background: Drop height has previously been used as an effective programming parameter in plyometric jump training. Less is known about the usage of maximal rebound jump height from a distinct drop height as a parameter for individualized plyometric jump training. Hence, the aim of this study was to contrast the effects of two different drop jump (DJ) training modalities using either the individualized maximal rebound height (MRHT) or a standard (SDHT) drop height on selected measures of physical fitness in young volleyball players. Methods: Thirty male young volleyball players aged 14 to 16 years were randomly assigned to an MRHT (*n* = 15) or an SDHT (*n* = 15) group. The MRHT group performed DJ exercises using a drop height according to the individual’s maximal rebound jump height from 30 cm, 40 cm, and 50 cm drop heights. The SDHT group performed DJs following a standardized drop height (30 cm) across the 8-week intervention period. The overall training volume was similar between MRHT and SDHT with one to three sets of 8 to 10 repetitions of DJ exercises per session. Before and after training, jump height and the reactive strength index (RSI) were taken as dependent variables from 30, 40, and 50 cm drop heights. In addition, dynamic balance (Y-balance test) as well as linear sprint and change-of-direction (CoD) speed were assessed. Results: Significant group × time interactions were found for jump height, balance, RSI, and linear sprint (*p* < 0.001; d = 0.12–3.42) but not CoD speed. Post hoc tests showed significant jump height improvements in favor of the MRHT group for drop heights from 30 cm (Δ20.4%, *p* < 0.001, d = 3.69), 40 cm (Δ20.3%, *p* < 0.001, d = 2.90), and 50 cm (Δ18.3%, *p* < 0.001, d = 3.37) and RSI50 (Δ30.14%, *p* < 0.001, d = 2.29). MRHT but not SDHT resulted in significant 5 m (Δ9.2%, *p* < 0.001, d = 1.32) and 20 m (Δ7.4%, *p* < 0.01, d = 2.30) linear sprint speed improvements. Conclusions: The findings demonstrate that MRHT but not SDHT improved DJ height, RSI, and linear sprint speed. Due to the importance of vertical jumps and short accelerations for overall competitive performance in volleyball, our results suggest that young male players should perform MRHT as part of plyometric jump training if the goal is to improve acceleration, reactive strength, and vertical jump performance.

## 1. Introduction

In volleyball, point-scoring actions (e.g., serve, spike, and block) are jump based, with a typical squad (*n*~12) of volleyball players performing ~120,000 jumps throughout a season [1]. Indeed, some investigations have demonstrated the beneficial effects of short-term [2,3] plyometric jump training (PJT) on measures of physical fitness in youth volleyball players. Similarly, dynamic balance is needed for spiking and blocking actions, which is why it has been considered an important prerequisite for successful volleyball performance [4,5]. Because volleyball players perform numerous jump-landing tasks during volleyball training and matches, jump performance and dynamic balance should be developed in youth volleyball players. Hence, jump-based exercises should be an integral part of a volleyball player’s strength and conditioning program.

As part of youth PJT, the drop jump (DJ) is an often-applied exercise. The DJ is a jump-landing exercise that is characterized by muscle actions in the stretch–shortening cycle (SSC) [6]. When performing DJs, the selection of appropriate drop heights is important for two reasons. First, adequate drop heights enable individuals to maximize training-induced physical and physiological adaptations and thus performance enhancement [7]. Second, adequate drop heights prevent excessive loads and thus mechanical stress on lower extremity muscle–tendon and bony structures [8,9]. Findings from cross-sectional studies indicate that drop heights influence DJ performance and neuromuscular activation [10,11]. For instance, Komi and Gollhofer [11] showed in their seminal study that the identification of an optimal drop height facilitates maximal rebound height. More specifically, if athletes perform DJs from drop heights beyond the optimal, muscle preactivation declines prior to ground contact, which has been interpreted as an inhibitory and protective mechanism aimed at preventing overload and injuries [10]. With lower muscle preactivation levels, stretch reflex contributions during ground contact are lower as well, which can be interpreted as a neuromuscular mechanism to prevent muscle-tendon injuries [10].

The results of the cross-sectional study by Prieske et al. [11] demonstrated progressively improved DJ performance (i.e., DJ height) with increasing drop heights from 20 cm to 35 cm and 50 cm in elite adolescent male and female handball players. Regarding findings from another cross-sectional study, Bassa et al. [12] reported no performance gains in DJ height with increasing drop heights in prepubertal children. Birat, et al. [13] reached similar conclusions based on findings from a cross-sectional study including pre-, circa, and post-pubertal youth. While the results from these cross-sectional studies [10,11,12,13] on the effects of drop height on DJ performance are interesting for plyometric training prescriptions with youth athletes, there remains a need to further examine appropriate methods on how to use maximal rebound height as a PJT programming parameter.

Using a longitudinal study approach, Taube et al. [7] found larger training-induced improvements in the reactive strength index (rebound height/duration of ground contact) (+14%) and soleus muscle activity in healthy adults aged 24 years. Ramirez-Campillo et al. [14] showed that the implementation of different maximal drop heights during PJT combined with regular soccer training significantly improved DJ rebound height, countermovement jump (CMJ) height, multiple five bounds, and maximal kicking distance performance but not linear sprint speed and maximal strength in youth soccer players compared with a group that performed soccer training only. Similarly, Ramirez-Campillo et al. [15] demonstrated that drop heights from 10 cm to 40 cm improved jump performance (CMJ: 17%; DJ: 36%), change-of-direction (CoD) speed (24%), reactive strength (5-repetition maximum [16]: 18%), and 20 m linear sprint time (24%) more than a standard drop height (i.e., 30 cm) during DJ training in youth soccer players. The authors concluded that the use of maximal drop heights during DJ training is suitable to maximize PJT effects in youth. So far, there is no study available that uses maximal rebound height as a method for programming DJ training. Of note, drop height refers to the height a performer falls from a box (e.g., 30 cm), whereas maximal rebound height refers to the maximum jump height achieved after landing from the box and immediately jumping off the ground [6]. In DJ training, if the drop height is too low, it may fail to sufficiently overload the neuromuscular system, thereby reducing the possible extent of adaptation [7]. In contrast, if the drop height is too great, the player may not be able to effectively control the eccentric and transition phases, thereby requiring the athlete to employ a technique that again does not sufficiently overload the neuromuscular system [7]. Hence, it is important to distinguish between drop height and maximal rebound height in understanding how maximal rebound height may be used as a tool for programming PJT. For example, an individual might undertake DJs from 20, 30, 40, and 50 cm boxes but achieve maximal rebound heights of 30 cm from the 20 cm box, 32 cm from the 30 cm box, 34 cm from the 40 cm box, and 28 cm from the 50 cm box.

Previously, researchers have reported that the jump strategy may impact jump load and intensity [17]. Researchers instructed athletes on the correct jump strategy and ensured, via observation and correction if needed, a consistent jump strategy. Moreover, there is a difference between drop height and actual fall height, which has multiple implications. Based on the results of Bobbert et al. [6], researchers are advised to limit dropping height to 20 or 40 cm when investigating the training effects of the execution of bounce drop jumps. Using the concept of maximal rebound height, the individual would exercise in this example using a 40 cm drop height because the maximal rebound height was achieved at a 40 cm drop height. For any given individual, maximal rebound height may differ; thus, training using the maximal rebound height reflects a person-centered and potentially optimal approach to PJT programming. Therefore, greater drop height during drop jump drills may have stimulated greater neuromuscular adaptations. From a mechanical perspective, the greater jumping height achieved may reflect greater participation of the SSC governing mechanisms (i.e., stretch reflex) [7], which are especially relevant in youths undergoing growth and maturation [18]. In this sense, greater SSC activity may be accompanied by greater muscle activation, including key muscle groups for players such as the medial gastrocnemius [19], thus contributing to the larger observed effects for physical fitness performance (i.e., drop height performance). However, it remains unresolved as to whether the effects of DJ training using the optimal drop height that produces a maximal rebound height versus a standard drop height (i.e., 30 cm) have similar or different effects on DJ performance.

Therefore, the objective of this study was to examine the effects of 8-weeks of DJ training using either the optimal drop height that causes maximal rebound height (MRHT) or standard (SDHT) drop height training on selected measures of physical fitness in highly trained young male volleyball players. In accordance with previous studies [7,14,15], we hypothesized that training-induced adaptation is more pronounced due to the individualized approach using the maximal jump rebound height (i.e., MRHT) approach.

## 2. Materials and Methods

### 2.1. Participants

The sample size was estimated using an a priori power analysis with a type I error rate of 0.05 and 80% statistical power. As a reference, we used the study of Ramirez-Campillo et al. [14]. The analysis indicated that 30 participants would be sufficient to observe a significant interaction effect (effect size Cohen’s f = 0.27 for DJ performance using a box height of 20 cm. Overall, 30 highly trained young male volleyball players from a national volleyball team (Esperance Sportive of Tunis, Tunis, Tunisia) were enrolled in this study (Table 1). The participants have performed systematic volleyball training in their club for the last 4 years with 4 to 5 weekly training sessions and a match played over the weekend. According to the classification of the expertise level provided by McKay et al. [20], the study sample can be categorized as Tier 3 (highly trained, national level). Based on previous PJT interventions, the athletes were familiar with PJT, including DJ exercises. Exclusion criteria included: (i) taking any dietary supplements during the study period, (ii) participation in additional non-team training, and (iii) adverse health events (i.e., injuries) that prevent the participants from performing drop jump training and physical fitness tests. Legal guardians and participants provided written informed consent and assent after a thorough explanation of the objectives and the scope of the research project, including the procedures, risks, and benefits of the study. The study was conducted according to the latest version of the Declaration of Helsinki, and the protocol was fully approved by the Local Clinical Research Ethics Committee “Personal Protection Committee” under the following code (N°: O220/2023) before the commencement of any assessments. Written informed consent was obtained from parents/legal representatives of all participants prior to the start of the study.

### 2.2. Procedures

The intervention was conducted during the first half of the season (September–October 2023). One week before the start of the study, a familiarization session was scheduled to allow young volleyball players to become acquainted with the applied tests and exercises. The testing protocol included the assessment of 30, 40, and 50 cm DJ boxes as dependent variables. In addition, the reactive strength index (RSI) from 30, 40, and 50 cm DJ boxes and dynamic balance were assessed using the Y-balance test and the composite score (CS-YBT). Furthermore, linear sprint speed was assessed over 5 and 20 m and CoD speed was assessed using the T-half test.

Before testing, a standardized warm-up was performed consisting of submaximal running for 5 min, 2–3 submaximal sprints over 10–15 m, and volleyball-specific exercises. Prior to strength testing, submaximal lower limb exercises (e.g., 10 squats, 3–5 countermovement jumps (CMJ)) were performed. All tests were separated by a 5–10 min rest period. The rest between the test trials was three minutes. The best out of the two trials was used for further statistical analyses. The same test sequence was applied during pre- and post-tests.

#### 2.2.1. Anthropometrics and Body Composition

Athletes’ body height and mass were collected using a wall-mounted stadiometer (Florham Park, NJ, USA) and an electronic scale (Baty International, West Sussex, England), respectively. The sum of skinfolds was assessed using Harpenden skinfold calipers. Anthropometric testing was conducted according to Deurenberg et al. [21] who reported similar prediction errors between adults and adolescents. Thereafter, biological maturity was evaluated non-invasively using chronological age and standing and sitting height as input parameters for a regression equation to subsequently predict the maturity offset [22]. The equation has previously been validated for boys and presents a standard error of estimate reported as 0.542 years [22].

#### 2.2.2. Drop Jump Performance

To assess DJ performance, participants performed maximal DJs from three drop heights (i.e., 20, 30, and 40 cm). DJ performance was measured using an Ergojump^®^ system (Ergojump, Globus Italia, Codogne, Italy). For the execution of DJs, participants stood in an upright position on the boxes, feet shoulder-width apart, with the hands placed on the hips. Participants were asked to step off the box with their dominant leg, drop down to land evenly on both feet (no heel contact allowed), and jump off the ground at maximal effort to perform a double-leg vertical jump. All participants were instructed to jump as high as possible and to keep the ground contact times as short as possible. Participants were asked to repeat the DJs when the ground contact times were >250 ms. However, no more than three DJ trials per drop height were performed. All participants reached ground contact times of <250 ms during two to four trials for all drop heights. Drop jump height as well as ground contact time were taken as dependent variables, and from these two measures, the RSI was determined. The RSI is the ratio of jump height and ground contact time. The best out of two trials (i.e., the highest RSI) was used for further analysis. Two DJ trials were completed for each height with a one-minute rest between jumps. The best out of the two trials was retained for further analysis.

#### 2.2.3. Dynamic Balance

Dynamic balance was evaluated using the CS-YBT [23]. All test trials were conducted barefoot. Participants stood on the dominant leg, with the most distal aspect of their big toe on the center of the footplate from the YBT kit. The participants were then asked to push the reach-indicator block with the free limb in the anterior, posterior medial, and posterior lateral directions while maintaining their single-limb stance on the central footplate [24]. CS-YBT data collection followed the protocol of Kang et al. [24]. Participants were not allowed to lift the heel of the stance leg during YBT performance. Maximal reach distances were recorded to the nearest 0.5 cm using the YBT kit.

#### 2.2.4. Linear Sprint Speed

The performance of a 5 m and 20 m linear sprint speed test was measured using photocell gates (Brower Timing Systems, Salt Lake City, UT, USA, accuracy of 0.01 s) placed 0.4 m above the ground. Split sprint times at 5 m and 20 m were analyzed. Participants started the test with one foot 50 cm before the starting line in an erect standing position and were instructed to accelerate as fast as possible. A starting signal was not provided in order to avoid the effect of reaction time. The rest period between single sprint trials amounted to ~1.5 min [25]. The best out of the two trials in terms of fastest sprint time was maintained for further analysis. The intra-class correlation coefficient for test-retest reliability for the 5 and 20 m sprint tests was 0.95 [3].

#### 2.2.5. Change-of-Direction Speed

COD speed was evaluated using the T-half test as previously outlined by Sassi, et al. [26]. The T-half test was used to determine the speed with directional changes such as forward sprinting, left and right shuffling, and backpedaling. Participants started the test with both feet behind the starting line. At their discretion, each participant had to sprint forward to a cone fixed at 5 m and touch the base of the cone with their right hand. Facing forward and without crossing their feet, they had to shuffle to the left to a cone fixed at 2.5 m and touch the base of the cone with their left hand. Subjects then had to shuffle 5 m to the right to the last cone and touch the base of the cone with their right hand. They had to shuffle again to the left to the first cone and touch the base of the cone. Finally, participants had to run backward as quickly as possible to return to the starting line. The total distance covered was 20 m. Any participants who crossed one foot in front of the other, failed to touch the base of the cone, and/or failed to face forward had to repeat the procedure. A total of three trials was performed by each participant with a 3 min rest in between trials. The best performance was recorded for further analysis.

#### 2.2.6. Drop Jump Training Programs

During the 8-week training intervention, the MRHT or SDHT groups replaced 20 min per session of the technical volleyball training content with DJs, performed twice a week separated by at least 48 h. Every participant received an ID number which was entered into an online randomization tool (https://www.randomizer.org/; accessed on 5 September 2023). Individuals were randomly allocated into two experimental groups. A detailed weekly description of the usual volleyball training applied during this period is shown in Table 2. Similar to other previously published training studies in athletic populations, in MacGawley and Andersson’s study [27], a passive control group could not be incorporated as the two experimental groups were national-level highly trained young players and there were no comparable players available that would provide similar baseline values. Previous studies have already demonstrated the effectiveness of plyometric and balance training in highly trained young players [3,28]. The objective of the present study was to compare the effects of two specific DJ training programs on measures of physical fitness in highly trained young volleyball players.

Participants in both intervention groups performed 1 to 3 sets of 8 to 10 repetitions of DJs per session, with 15 and 90 s of rest between repetitions and sets [29], respectively. Overall training volumes were similar between MRHT and SDHT.

The MRHT group performed DJ exercises using a drop height according to the individual’s maximal rebound jump height from 30 cm, 40 cm, and 50 cm drop heights. For this purpose, participants performed DJs from different drop heights (30, 40, and 50 cm), and the drop height was used for training where the athletes achieved the largest rebound height. For example, if the player achieved their best rebound height from a drop height of 40 cm, they would use a rebound height of 40 cm during training. The tests to establish a maximal rebound height were repeated on a weekly basis to allow training progression). During the pre-test, 12 participants achieved their maximal rebound height at 30 cm drop height, 2 participants achieved their maximal rebound height at 40 cm drop height, and no participant achieved their maximal rebound height at 50 cm drop height.

The SDHT group performed DJs following a standardized drop height (30 cm) across the 8-week intervention period. Of note, 30 cm drop height is a frequently applied and recommended drop height for drop jump training in young athletes. Across the 8-week intervention period, the standardized drop height group performed 1 to 3 sets of 8 to 10 repetitions of DJs per session, with 15 and 90 s of rest between repetitions and sets. In other words, progression was achieved through the manipulation of these training load variables and not through the adjustment of drop height.

Participants in the MRHT and SDHT were asked to aim for maximal rebound height and minimal contact time during each jump. Throughout all exercise sessions, the instructor-to-player ratio of 1:1 was maintained. The RPE was adjusted every two weeks using a 0–10 OMNI scale. At the start of the intervention, an RPE of three on the OMNI scale was targeted. During weeks 3–4, the RPE was increased to five on the OMNI scale. During weeks 5 to 6, the RPE was increased to 7. In weeks 7–8, an RPE of nine was targeted.

Before the start of MRHT or SDHT, participants completed a standardized warm-up consisting of low-intensity jogging, CoD, DJ, and dynamic lower-limb stretching.

### 2.3. Statistical Analyses

All data analyses were performed using SPSS 26.0 (SPSS, Inc., Chicago, IL, USA). The level of significance was set a priori at *p* < 0.05. Data were tested and confirmed for normal distribution using the Shapiro–Wilk test. Subsequently, a 2 (groups: MRHT, SDHT) × 2 (time: pre and post) analysis of variance (ANOVA) was computed with repeated measures on time to establish the exercise effects of the different DJ training programs on measures of physical fitness. If group-by-time interactions reached the level of significance, group-specific post-hoc tests (i.e., Bonferroni corrected pairwise comparisons) were computed to identify the comparisons that were statistically significant. Additionally, effect sizes were determined by converting partial eta-squared from the ANOVA output to Cohen’s d [30]. Cohen’s d was classified as small (0.00 ≤ d ≤ 0.49), medium (0.50 ≤ d ≤ 0.79), and large (d ≥ 0.80). Reliability of the assessed variables was computed using Cronbach’s model of ICCs(3.1) and standard error of measurements (SEM) according to the method introduced by Hopkins et al. [31]. We considered an ICC below 0.40 as poor, between 0.40 and 0.70 as fair, between 0.70 and 0.90 as good, and >0.90 as excellent. The SEM was calculated by dividing the SD of the difference between scores by √2. The SEM allows the calculation of the minimal detectable change at the 95% CI (MDC95) according to the following formula: MDC = SEM*1.96* √2. Recognizing the need to present individual response data in studies where training interventions take place, especially where there are sample sizes at the level of the present study, we followed the guidelines of Weissgerber et al. [30,32] and present figures showing the mean and individual response data for each condition. Such data visualization offers the opportunity to observe all of the data available.

## 3. Results

All 30 highly trained young male volleyball players completed the study and took part in the interventions as allocated. The adherence rate was above 85% for all participating athletes. Hence, four participants did not complete the study and were therefore excluded. No test- or training-related acute or overuse injuries were reported during the study that needed medical guidance. In addition, no significant between-group baseline differences were observed for all analyzed variables (Table 1). Table 3 displays the test–retest reliability data for all assessed variables.

### 3.1. Drop Jump Performance

**Figure 1:**
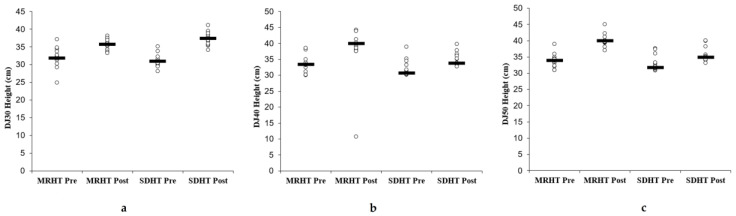
Individual and mean pre- and post-test data of drop height from a 30 (**a**), 40 (**b**), and 50 cm (**c**) drop height according to the experimental groups; drop height 30 cm: drop height from a box of 30 cm;; MRHT: drop jump training using an individual maximal rebound jump height; SDHT: drop jump training using a standard box with a 30 cm drop height.

Our results revealed a significant effect of time for the DJ-30, DJ-40, and DJ-50 tests (*p* < 0.005, *p* < 0.001, and *p* < 0.001; d = 0.77, 2.18, and 2.26, respectively). In the same context, significant group × time interactions were found for DJ-30, DJ-40, and DJ-50 cm (*p* < 0.05, *p* < 0.002, and *p* < 0.002; d = 0.54, 0.85, and 0.88, Figure 1). Group-specific post hoc analyses showed a large performance improvement in DJ 30 cm for SDHT (∆20.4%, *p* < 0.001, d = 3.69). In addition, large performance improvements were found for the DJ-40 and DJ-50 cm condition (∆20.3% and 18.3%, both *p* < 0.001, d = 2.90 and 3.37, respectively) for the MRHT group.

### 3.2. Dynamic Balance

A significant main time effect was found for the CS-YBT (*p* < 0.003, d = 0.82). However, the group × time interaction failed to reach the significance level (*p* = 0.623, d = 0.12).

### 3.3. Linear Sprint Speed

A significant main time effect was found for both the 5 m (*p* < 0.002, d = 0.89) and the 20 m (*p* < 0.001, d = 1.56) sprint performance tests (Table 4). Our statistical calculation showed a tendency towards a significant group × time interaction for the 5 m sprint test (*p* < 0.067, d = 0.50). The post hoc analysis indicated a large improvement in 5 m sprint performance from the pre- to post-test in favor of the MRHT group (∆9.2%, *p* < 0.001, d = 1.32). The 20 m sprint test displayed a significant group × time interaction (d < 0.77, *p* < 0.005). The post-hoc analysis showed a large improvement in 20 m sprint performance from the pre- to post-test in favor of the MRHT group (∆7.4%, *p* < 0.001, d = 2.30).

### 3.4. Change-of-Direction Speed

A significant main effect of time (*p* < 0.001, d = 1.7) was found for the T-half test. However, the group × time interaction failed to reach the significance level (*p* = 0.101, d = 0.22).

### 3.5. Reactive Strength

Our statistical calculation revealed a significant main time effect (d = 4.24, 1.47, and 3.42, respectively for the RSI30, RSI40, and RSI50; all *p* < 0.001). Likewise, a significant group × time interaction was found for the RSI50 (*p* < 0.001; d = 1.19). The post-hoc analysis showed a large improvement in RSI50 performance from the pre- to post-test in favor of the MRHT group (∆30.14%, *p* < 0.001, d = 2.29). However, no significant group × time interactions were noted for the RSI30 and RSI40 (all *p* > 0.05). In regards to the contact time parameters, the statistical analysis indicated significant main effects of time (all *p* < 0.001, d = 1.96, 2.07, and 2.08 for the CT30, CT40, AND CT50, respectively). However, no significant group × time interactions were found (all *p* > 0.05).

## 4. Discussion

To the authors’ knowledge, there is no study available on this topic dealing with the effects of two DJ training protocols using MRHT vs. SDHT on selected measures of physical fitness in highly trained young male volleyball players. When performing DJ training, drop height is considered a key variable in adjusting training intensity and the respective performance output [33]. The main result of this study was that MRHT demonstrated larger vertical jump height, reactive strength, and sprint performance improvements than SDHT. Exercise-induced improvements in dynamic balance and CoD speed were similar in both groups. The results of the current study provide insights for coaches and athletes in regard to using MRHT as a programming variable for performance development in highly trained young male volleyball players.

The findings indicate that MRHT resulted in larger performance improvements for DJ performance compared with SDHT. In addition, our statistical calculation showed that the change percentage from the pre-test to post-test was greater than the SEM in both groups.

The greater improvement in the MRHT group may be related to the use of an optimal and specific box drop height during maximal-effort vertical drop jumps, leading to greater jumping and RSI development [15]. For highly trained young male volleyball players (Tier 3) aged 24 years, Andrade et al. [19] showed that DJs from a 40 cm drop height induced RSI improvements of 22% compared to a 20 cm drop height. Further, Ramirez-Campillo et al. [14] showed a moderate improvement in DJ performance using a drop height of 40 cm compared with a 20 cm drop height on measures of DJ performance (ES = 0.20–0.55; *p* < 0.05) in youth soccer players. Previous cross-sectional [10,11] and longitudinal [7,14,15] studies have already shown that beyond an optimal drop height, muscle pre-activation declines prior to ground contact, which has been interpreted as an inhibitory mechanism aimed at preventing injury. In terms of drop height, it has been suggested that youth athletes may be able to use SSC capabilities to increase vertical jump performance [12]. Specifically, the ability to utilize the SSC is pivotal to storing elastic energy during eccentric muscle actions and then releasing it to produce forceful concentric contractions [34]. In addition, greater leg muscle activities during preactivation appear to increase muscle stiffness by preparing agonist muscles to better resist high-impact loads due to increased drop heights [8,11]. In this context, Ramirez-Campillo et al. [14] showed that an optimal drop height (i.e., 10 cm to 40 cm: generating an optimal RSI) improved jump performance (CMJ: 16.7%, d = 0.76; DJ: 36.1%, d = 0.79), CoD-speed (24.4%, d = 20.34), reactive strength (5RM: 18.1%, d = 0.47), and 20 m sprint time (23.7%, d = 0.27) more than a standard drop height (i.e., 30 cm) during DJ training in highly trained young soccer players (Tier 3). The authors concluded that the use of maximal drop heights during DJ training is a meaningful tool to maximize the effects of plyometric jump training in youth. Nevertheless, the underlying mechanisms leading to potentially greater neuromuscular adaptations after MRHT compared to SDHT are related to the potentially greater intensity achieved with higher/optimal rebound heights [7]. Therefore, greater DJ rebound height may have stimulated greater neuromuscular adaptations. Moreover, greater DJ height achieved may reflect greater participation of the SSC governing mechanisms (i.e., stretch reflex) [7], which are especially relevant in youths undergoing growth and maturation [18]. In this sense, a larger SSC may be accompanied by greater muscle activation, including key muscle groups such as the medial gastrocnemius and soleus [19], thus contributing to the better vertical jump performance observed for DJ at 30, 40, and 50 cm. Therefore, the prescription of maximal effort during DJs according to the maximal rebound height may enable meaningful physiological adaptations in highly trained young volleyball players (Tier 3). An important question that might be asked in relation to the results presented in the current study is whether substantial physiological improvements can be expected in an eight-week training period with highly trained young athletes. The available literature on this topic suggests that neuromuscular adaptations can be observed after approximately four weeks of drop jump training in highly trained athletes [7,35]. As a consequence, in the present study, we would suggest that the training period employed is sufficient to elicit neuromuscular adaptations that translate to improved performance. The underpinning pathways for such changes are likely due to increased pre-activation of the lower limb musculature (m. gastrocnemius), where feed-forward control of the neuromuscular system (i.e., pre-programmed muscle activation) results in subsequent feedback action control in the form of a stretch reflex to pre-activate the muscles in anticipation of the reaction force that the joint will be subjected to and forms appropriate muscle stiffness. It is likely that DJ rebound height elicits a greater stretch reflex, resulting in greater pre-activation of lower limb muscles, increased muscle stiffness, and thus better performance. The results of this study indicate that MRHT has a favorable effect on acceleration (5 m [Δ9.2%, *p* < 0.001, d = 1.32]) and linear sprint speed ([20 m (Δ7.4%, *p* < 0.01, d = 2.30]) in highly trained young volleyball players. Likewise, the findings revealed that the percentage changes from the pre-test to post-test in the 5 m and 20 m tests were greater than the SEMs in the MRHT group. However, in the SDHT group, the percentage changes in the 5 and 20 m tests failed to exceed the SEMs. Thus, training-related gains in vertical DJ performance (e.g., maximal rebound height) may partly translate to the observed improvements in short linear sprint speed in young athletes.

A possible reason for this may be the individualized maximal rebound heights used during the MRHT which could result in enhanced muscle stretch tolerance during the amortization phase of DJ drills in youth [36]. Another explanation could be related to the better utilization of reactive strength (i.e., RSI) [14]. In this context, it can be postulated that a higher rebound height during MRHT demands longer ground contact times which might be beneficial for better transference to the acceleration phase of running when compared to the high-speed running phase. Accordingly, a study conducted by Loturco et al. [37] with a sample of soccer players under 20 (mean age, ~18 years) compared sprint training adaptation of a group that trained for three weeks with either vertical jumps (i.e., CMJ) or horizontal jumps. In the vertically trained group, the 20 m sprinting speed was 1.31, and for acceleration in 10–20 m, it was 2.75. In the horizontally trained group, the 10 m sprinting speed was 0.44, the 20 m sprinting speed was 0.17, and for acceleration in 0–10 m, it was 0.44. Moreover, when highly trained young male volleyball players under the age of 20 (mean age, ~18 years) trained for 6 to 8 weeks (Tier 3) [20] with either DJ squat or push-press exercises at the optimum power load [37], a meaningful difference in acceleration and maximal speed performance was detected only for those players who trained with MRHT. In this regard, it was suggested that both DJ and sprint performances at longer distances essentially rely on the SSC paradigm, whereas shorter sprint distances, where acceleration is greatest, are more related to maximum strength (e.g., half squat) [15]. Nonetheless, to date, no study has examined the transference effects of DJ training prescription with respect to a more comprehensive variety of fitness attributes in volleyball.

Researchers may consider assessing the optimal rebound of the trained DJ exercises by including a test battery with such trained exercises among the dependent variables. Although horizontal force orientation is paramount for sprint performance, the application of force and power in the vertical axis is also practically significant for sprinting speed [15]. In this sense, it is interesting to hypothesize that given the use of MRHT vertical-oriented RSI during training in the MRHT group, this may have induced meaningful sprinting adaptations, leading to better utilization of reactive strength during sprinting (i.e., 5 m acceleration), particularly during the vertical application of force and power.

A similar improvement in dynamic balance following MRHT is in accordance with previous PJT interventions conducted in highly trained young athletes [38]. In addition, the findings revealed that performance changes from the pre-test to post-test were greater than the SEMs in both groups. Using both MRHT and SDHT as a dynamic form of PJT [39], the specificity of the exercises would induce training stress on stability [40] by promoting anticipatory postural adjustments [41]. In addition, while PJT exercise is prevalent in volleyball training, we conceptualized that both MRHT and SDHT were performed with increased-speed, dynamic contractions performed within a smaller base of support or with the center of gravity being moved outside the base of support [7,40]. As a consequence, both MRHT and SDHT exercises are likely to elicit a similar improvement in balance performance in highly trained young volleyball players.

Furthermore, our study reports similar improvements in CoD speed (T-half-test) for MRHT and SDHT in highly trained young male volleyball players. The findings showed that the percentage change from the pre-test to post-test was greater than the SEM (Table 3). Several PJT studies have reported the use of a maximal drop height in order to increase the intensity-based load [11,14,15]. Similarly, the rebound achieved during the MRHT was also progressively increased during training (from 20 to 50 cm) which can lead to a similar improvement in some outcomes (i.e., CoD speed). These results are in accordance with previous studies [15,38]. As the MRHT and SDHT exercises were used to identify the different PJT intensities, these training regimens may similarly improve the eccentric strength of the lower limbs, resulting in increased CoD performance [38]. Also, neural adaptations in the form of better motor unit recruitment may represent other mechanisms that can lead to increased CoD speed [38]. From a mechanical perspective, the greater jump height achieved in both DJ training groups may reflect greater contributions of the stretch-reflex [7], which are especially relevant during growth and maturation [18]. However, with our methodological approach, we could not determine neuromuscular adaptations. Future studies are needed to determine the underlying physiological mechanisms responsible for exercise-induced CoD improvements following DJ training in highly trained young volleyball players.

This study is not without limitations. First, we did not include a passive control group in the current study due to ethical reasons. Given the population participating in the study, i.e., youth athletes, it would be ethically questionable to refrain youth athletes from a certain training regime. Consequently, the outcomes of the present study have to be interpreted with consideration of the nature of the control group examined. In addition, higher drop heights in SDHT may further increase DJ height but at the expense of longer ground contact time and thus DJ technique. Hence, in terms of the jump strategy, the drop jump test instructions should focus on the proper movement technique to make sure that the jump strategy does not change. It is also important to note that participants who trained at a position equal to or higher than the standard 30 cm showed better results. This difference may not solely be because the training volume is not the same, but also because the training intensity (i.e., drop height) is different. Separating training volume and intensity in the context of drop jump training is practically difficult without employing a complex research design with a considerable number of participants. This however is not feasible in the context of elite sports due to a low inherent number of available athletes. Hence, researchers and practitioners should be mindful of this possibility when interpreting the results of the present study. Furthermore, we were unable to examine the underlying neuromuscular mechanisms responsible for the observed changes in measures of physical fitness due to the lack of including neurophysiological testing apparatus (e.g., surface electromyography, isokinetic strength and power testing, etc.). Therefore, future studies are advised to include electrophysiological testing apparatus (e.g., electromyography) to elucidate the underlying neural changes.

In youth, growth and maturation are two important moderators of exercise effects. Therefore, future studies should evaluate the two training programs according to their maturity status (e.g., pre-pubertal vs post-pubertal athletes). In addition, the possibility of isolating the “trained exercises” (i.e., drop jumps) and determining their transfer effects with respect to some important volleyball-specific capacities has crucial importance for the programming of training interventions. Based on our results, strength and conditioning coaches working with highly trained young volleyball players should consider implementing MRHT programs using drop heights that produce maximal rebound height to enhance vertical jump performance, reactive strength, and short sprint time.

## 5. Conclusions

This study examined the effects of two drop jump (DJ) training modalities using maximal rebound height (MRHT) vs standard (SDHT) drop height programming approaches on selected measures of physical fitness in highly trained young male volleyball players. Due to the importance of vertical jumps and short accelerations for overall competitive performance in volleyball, our results suggest that highly trained young male volleyball players should perform MRHT as part of PJT if the goal is to improve acceleration, reactive strength, and vertical jump performance.

As muscle power and sprint performance are directly related to success in volleyball competition [42], the results of the present study might be considered to be implemented in regular youth volleyball training. Coaches should be mindful of the results of the present study when working with highly trained young male volleyball players and applying PJT. Therefore, if the objective is to enhance jump performance, reactive strength, and short linear sprint speed in highly trained young male volleyball players, MRHT should be applied.

While our study sample consisted of experienced highly trained young male volleyball players, the findings of this study should be compared with those of novice players. It seems plausible that SDHT exercises could be more appropriate for less experienced volleyball players due to lower training loads during DJ training. Finally, although plyometric training can induce an increase in measures of physical fitness in highly trained young volleyball players, to optimize training adaptations, this training strategy should be adequately applied in a more complex training plan that incorporates other explosive (e.g., sprints), power, technical, and tactical-oriented training methods.

## Figures and Tables

**Table 1:** Anthropometric characteristics of the participating male volleyball players at baseline.

Variables	MRHT Group (*n* = 15)	SDHT Group (*n* = 15)	*p*-Value
Age (years)	15.88 ± 0.48	15.33 ± 0.57	0.38
Height (cm)	185.67 ± 6.15	182.60 ± 0.93	0.14
Body mass (kg)	72.53 ± 10.08	69.20 ± 7.83	0.32
Maturity offset (years)	2.65 ± 0.48	2.11 ± 0.50	0.005
APHV (years)	13.23 ± 0.39	13.22 ± 0.45	0.95

Note: Values shown as the means ± standard deviations; APHV: age at peak height velocity; MRHT: drop jump training using an individual maximal rebound jump height; SDHT: drop jump training using a standard box 30 cm drop height.

**Table 2:** Representative microcycle applied during the study period.

Monday	Tuesday	Wednesday	Thursday	Friday	Saturday	Sunday
Warm-up (15 min) MRHT and SDHT (20 min) Tactical defensive situations (20 min) Simulated matches (30 min) Cool down (5 min)	Warm-up (15 min) Technical passing drills (20 min) Machine-based contrast strength training) (20 min) Simulated matches (30 min) Cool down (5 min)	Warm-up (15 min) Technical smashing and passing drills (20 min) Vertical and horizontal countermovement jump (20 min) Simulated matches (30 min) Cool down (5 min)	Warm-up (15 min) MRHT and SDHT (20 min) Tactical offensive situations (20 min) Simulated matches (30 min) Cool down (5 min)	Warm-up (15 min) Technical spiking and passing drills (20 min) Tactical offensive and defensive situations) (20 min) Simulated matches (30 min) Cool down (5 min)	Competition	Day off
90 min	90 min	90 min	90 min	90 min		

Notes: PJT: plyometric jump training; MRHT: drop jump training using the drop height that produced the maximal rebound jump height; SDHT: drop jump training using a standard box 30 cm drop height.

**Table 3:** Test–retest reliability of the applied balance, power, sprint, and change-of-direction speed tests.

Variables	ICC (3.1) (95% CI)	SEM	CV (%)
Dynamic balance			
CS-YBT (°/°)	0.89 [0.81–0.91]	1.22	1.74
Vertical jump height			
DJ height			
DJ 30 cm (cm)	0.91 [0.88–0.93]	0.96	2.14
DJ 40 cm (cm)	0.90 [0.87–0.92]	0.91	2.17
DJ 50 cm (cm)	0.89 [0.78–0.90]	1.92	1.13
Contact time			
CT 30 cm (ms)	0.87 [0.73–0.90]	0.73	4.23
CT 40 cm (ms)	0.91 [0.90–0.96]	0.80	1.65
CT 50 cm (ms)	0.90 [0.87–0.93]	0.15	2.02
Reactive strength			
RSI 30 (cm)	0.90 [0.83–0.94]	4.38	1.06
RSI 40 (cm)	0.92 [−0.89–0.96]	6.62	4.36
RSI 50 (cm)	0.91 [0.88–0.97]	0.46	4.78
Linear sprint speed			
5 m (s)	0.91 [0.82–0.96]	3.41	1.32
20 m (s)	0.92 [0.87–0.93]	2.17	2.16
Change-of-direction speed			
T-half-test (s)	0.88 [0.81–0.90]	1.81	2.12

Notes: ICC (3.1) intra-class correlation coefficient, CI confidence interval, SEM standard error of measurement, CS-YBTRL: composite score during the Y-balance test with the dominant leg, DJ 30 cm: drop height 30 cm: DJ from a box of 30 cm, CoD: change-of-direction, RSI: strength reactive index, and CT: contact time.

**Table 4:** Effects of MDJT and FDJT on measures of physical fitness in youth male volleyball players.

Variables	Groups	Pre-Intervention	Post-Intervention	Δ Change %		ANOVA *p*-Value (Cohen’s d)		
					Cohen’s d (d Lower Limit to d Upper Limit)	Time	Group	Group × Time
Vertical jump performance								
	SDHT	32.01 ± 2.76	33.12 ± 9.26	3.47	−0.17 (−1.56 to 4.52)	0.005 (0.78)	0.184 (0.36)	0.046 (0.54)
DJ 30 cm	MRHT	31.11 ± 1.69	37.47 ± 1.85	20.44	−3.69 (−4.55 to −2.75)			
DJ 40 cm	SDHT	32.07 ± 2.44	35.05 ± 1.95	9.27	−1.40 (−2.63 to −0.41)	0.001 (2.18)	0.001 (1.44)	0.002 (0.85)
	MRHT	33.39 ± 2.54	40.17 ± 2.30	20.33	−2.90 (−4.18 to −1.73)			
DJ 50 cm	SDHT	32.94 ± 2.26	35.66 ± 2.09	8.26	−1.29 (−2.44 to −0.24)	0.001 (2.26)	0.001 (1.37)	0.002 (0.88)
	MRHT	33.90 ± 2.01	40.12 ± 1.80	18.34	−3.37 (−4.39 to −2.46)			
Dynamic balance								
CS-YBT	SDHT	103.49 ± 7.58	108.77 ± 8.33	5.10	−0.69 (−4.52 to3.53)	0.003 (0.82)	0.999 (0.001)	0.623 (0.12)
	MRHT	102.48 ± 7.94	109.79 ± 8.04	7.14	−0.95 (−4.97 to 3.12)			
Linear sprint speed								
5 m sprint (s)	SDHT	1.44 ± 0.08	1.40 ± 0.10	2.55	0.46 (0.42 to 0.51)	0.002 (0.89)	0.003 (0.82)	0.067 (0.50)
	MRHT	1.41 ± 0.08	1.28 ± 0.12	9.25	1.32 (1.28 to 1.38)			
20 m sprint (s)	SDHT	3.68 ± 0.13	3.59 ± 0.11	2.45	0.77 (0.71 to 0.83)	0.001 (1.56)	0.001 (1.46)	0.005 (0.77)
	MRHT	3.60 ± 0.10	3.33 ± 0.14	7.39	2.30 (2.25 to 2.37)			
Change-of-direction speed								
T-half test (s)	SDHT	6.32 ± 0.14	5.94 ± 0.31	5.97	1.64 (1.56 to 1.79)	0.001 (1.7)	0.094 (0.45)	0.101 (0.22)
	MRHT	6.31 ± 0.25	5.67 ± 0.46	10.22	1.79 (1.66 to 2.02)			
Reactive strength								
	SDHT	1.57 ± 0.13	1.92 ± 0.08	22.06	−3.36 (−3.42 to −3.32)	0.001 (4.24)	0.536 (0.17)	0.145 (0.40)
RSI30	MRHT	1.52 ± 0.07	1.94 ± 0.08	27.52	−5.78 (−5.82 to −5.74)			
RSI40	SDHT	1.56 ± 0.13	1.81 ± 0.10	16.14	−2.23 (−2.30 to −2.18)	0.001(1.47)	0.014 (0.68)	0.182 (0.36)
	MRHT	1.63 ± 0.13	2.05 ± 0.42	25.53	−1.10 (−1.46 to −1.19)			
RSI50	SDHT	1.60 ± 0.10	1.84 ± 0.10	15.14	−2.48 (−2.53 to −2.43)	0.001(3.42)	0.001 (1.71)	<0.001(1.19)
	MRHT	1.66 ± 0.11	2.16 ± 0.30	30.14	−2.29 (−2.35 to −2.14)			
CT30	SDHT	205.08 ± 14.57	193.30 ± 2.51	5.75	1.17 (−6.21 to 2.44)	0.001 (1.96)	0.031 (0.59)	0.149 (0.39)
	MRHT	203.59 ± 2.10	185.93 ± 4.26	8.67	4.82 (3.76 to 4.82)			
CT40	SDHT	206.12 ± 14.57	193.47 ± 2.51	6.14	1.25 (−6.12 to 2.52)	0.001 (2.07)	0.031 (0.59)	0.149 (0.39)
	MRHT	204.63 ± 2.10	186.10 ± 4.26	4.26	5.71 (4.65 to 7.87)			
CT50	SDHT	206.24 ± 14.57	193.57 ± 2.51	6.15	1.25 (−6.12 to 2.52)	0.001 (2.08)	0.031 (0.59)	0.149 (0.39)
	MRHT	204.75 ± 2.10	186.20 ± 4.26	4.26	5.72 (4.65 to 7.87)			

Notes: CS-YBT: composite score during the Y-balance test with the dominant leg; drop height 30 cm: DJ from a box with a 30 cm drop height; MRHT: drop jump training using an individual maximal rebound jump height; SDHT: drop jump training using a standard box with a 30 cm drop height; RSI: strength reactive index; CT: contact time.

## Data Availability

The datasets generated and/or analyzed during the current study are not publicly available. Upon reasonable request, the corresponding author will share the dataset.
